# Hypothesis: platelet-rich plasma as an adjunct therapy for eczema targeting inflammation, skin barrier repair, and chronic recurrence

**DOI:** 10.3389/fimmu.2025.1692916

**Published:** 2025-10-30

**Authors:** Ju Tian, Jing Ding, Huimin You, Chunhui Ou, Hongyuan Zhu, Biao Cheng

**Affiliations:** ^1^ Department of Plastic Surgery, Zhongshan City People’s Hospital, Zhongshan, Guangdong, China; ^2^ Skin Care Unit, Zhongshan City People’s Hospital, Zhongshan, Guangdong, China; ^3^ Department of Plastic Surgery, Affiliated Hospital of Guangdong Medical University, Zhanjiang, Guangdong, China; ^4^ Department of Plastic Surgery, General Hospital of Southern Theater Command, People’s Liberation Army, Guangzhou, Guangdong, China

**Keywords:** eczema, platelet-rich plasma (PRP), atopic dermatitis (AD), barrier repair, immunomodulation, antimicrobial activity, chronic inflammation

## 1 Introduction

Eczema comprises a group of chronic, relapsing inflammatory skin disorders characterized by erythema, papules, vesicles, exudation, and pruritus, and includes subtypes such as atopic dermatitis (AD), contact dermatitis, nummular eczema, and asteatotic eczema (1–3). The core pathological features consist of pruritus, barrier dysfunction, and immune dysregulation. Although treatment options have improved, current therapeutic strategies remain unsatisfactory: prolonged use of topical corticosteroids can cause skin atrophy, while calcineurin inhibitors such as tacrolimus show limited efficacy in certain patients (4, 5). Biologics like dupilumab and JAK inhibitors are effective for moderate-to-severe cases but present challenges including high cost, the need for injection, and potential risks of infection or malignancy (5, 6).

The pathogenesis of eczema arises from complex interactions among genetic susceptibility (e.g., filaggrin (FLG) mutations), impaired skin barrier function (e.g., elevated pH and microbial dysbiosis), dysregulated immune responses (including Th2/Th17 imbalance), and environmental factors (1, 4, 7, 8). This multifactorial complexity complicates the development of targeted therapies; biologics directed at specific pathways, such as Th2, often fail to ameliorate concurrent barrier impairment or microbial imbalance (9, 10). These limitations highlight the need for novel, safe, and multifaceted treatment strategies.

Platelet-rich plasma (PRP), an autologous blood product containing concentrated platelets and bioactive molecules including platelet-derived growth factor (PDGF), vascular endothelial growth factor (VEGF), Epidermal Growth Factor (EGF), Insulin-like Growth Factor (IGF) and transforming growth factor-β(TGF-β), has gained attention as a potential therapeutic agent in dermatology (11).

In addition, PRP also exhibits anti-apoptotic effects, immunosuppressive properties, reduction of melanin synthesis, antimicrobial activity, and antioxidant effects (11). Clinically, PRP has been demonstrated to be effective in treating various dermatological conditions, including androgenetic alopecia (by stimulating hair follicle stem cells and reducing perifollicular inflammation) (12), alopecia areata (AA) (13) and psoriasis (through normalizing keratinocyte hyperproliferation and downregulating the pro-inflammatory interleukin (IL)-17, a Th17-related cytokine) (14).

Notably, both AA and psoriasis share pathophysiological overlaps with eczema: all involve chronic immune activation (e.g., Th2/Th17 skewing), epithelial barrier disruption, and neuroimmune crosstalk (15–18). These conserved pathways imply that the multifunctional properties of PRP, namely anti-inflammatory, barrier-repairing, and immunomodulatory effects, may similarly target core mechanisms in eczema. To our knowledge, no systematic studies have yet explored the use of PRP in eczema treatment, and available data regarding its role in the condition’s complex pathophysiology remain limited. Considering the unmet clinical need and the favorable safety profile of PRP, we hypothesize that PRP could act as a novel adjuvant therapy for eczema, potentially mitigating inflammation, restoring epidermal barrier function, and reducing chronic recurrence via its pleiotropic effects. This study seeks to elucidate the mechanisms through which PRP modulates eczema, thereby providing preclinical evidence to inform future clinical trials and potentially advance toward more personalized and effective management strategies.

## 2 The hypothesis

**Figure 1:**
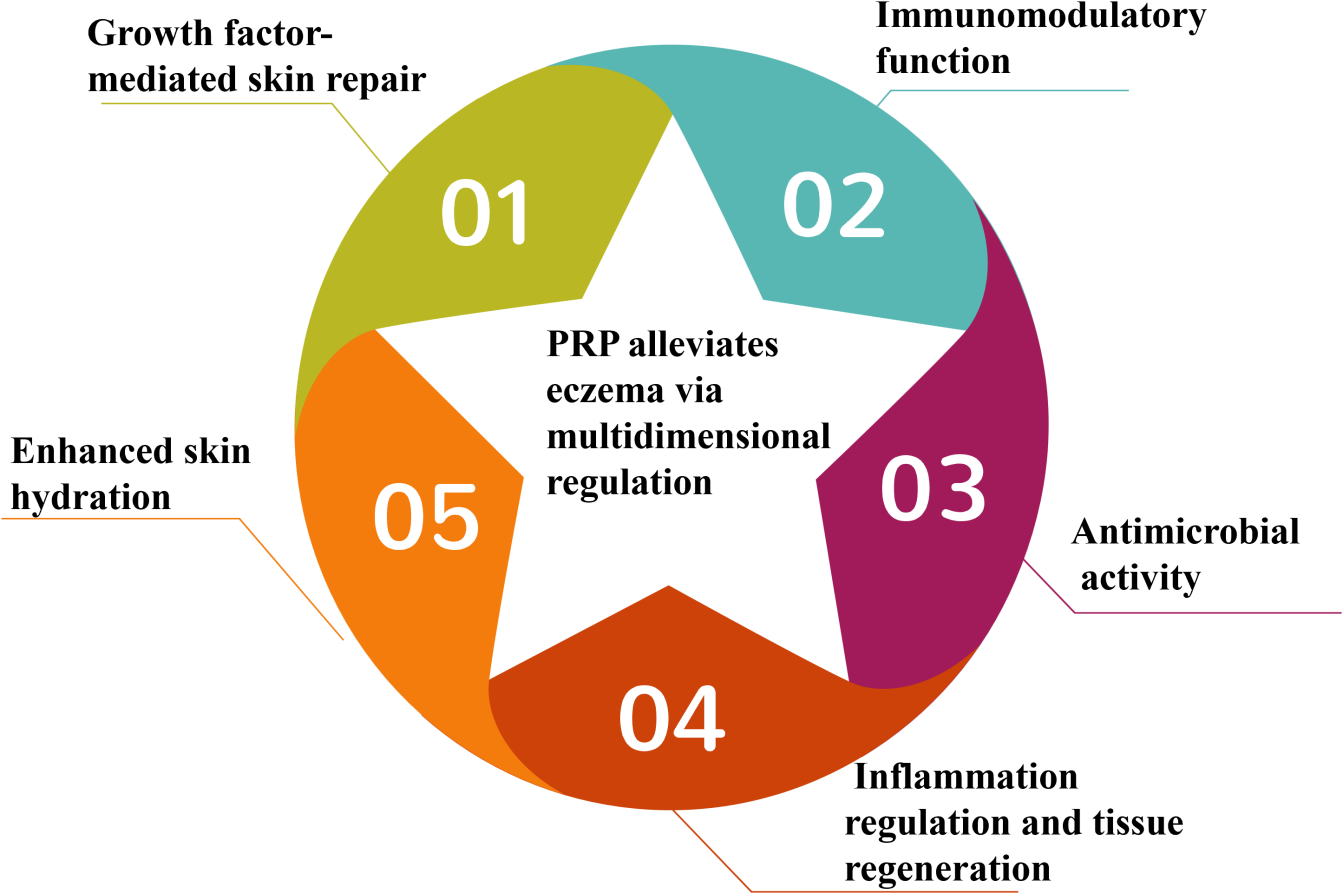
The multidimensional regulatory hypothesis and core mechanisms of PRP in treating eczema.

We hypothesize that PRP, as a novel adjuvant therapy for eczema, may enhance disease management through multidimensional regulatory mechanisms by alleviating inflammation, restoring epidermal barrier function, and reducing chronic recurrence via its pleiotropic biological effects (**Figure 1**). This synergistic interaction between PRP and affected skin is mediated by five core mechanisms:

### 2.1 Growth factor-mediated skin repair

PRP is enriched with diverse growth factors—such as TGF-β, PDGF, EGF, IGF, and VEGF—that promote skin regeneration and repair. These factors stimulate keratinocyte proliferation, enhance angiogenesis, and facilitate extracellular matrix (ECM) formation, all essential for repairing barrier damage in eczema. Experimental studies indicate that PRP increases the antioxidant capacity of keratinocytes and modulates melanin content, suggesting a potential role in regulating eczema-related skin inflammation and pigmentary changes (19).

### 2.2 Immunomodulatory function

PRP may ameliorate immune dysregulation in eczema by modulating T cell-related cytokines (13). It has also demonstrated significant analgesic effects in neuropathic pain (20); since neuroimmune interactions drive pruritus in eczema, PRP may alleviate symptoms through similar pathways.

### 2.3 Antimicrobial activity

Staphylococcus aureus (S. aureus) colonization or infection frequently occurs in eczematous skin and exacerbates disease progression. PRP may counteract this through direct antimicrobial actions (21) or by enhancing host defense mechanisms, thereby reducing infection-triggered flare-ups.

### 2.4 Inflammation regulation and tissue regeneration

Platelets in PRP release bioactive molecules that regulate inflammation, wound healing, and immune responses (22). These mechanisms may also operate in chronic eczema-related skin damage, facilitating the recovery of persistent lesions.

### 2.5 Enhanced skin hydration

Although direct moisturizing effects of PRP lack strong evidential support, its ability to restore barrier function may indirectly reduce transepidermal water loss (TEWL), thereby improving the overall hydration status of the skin.

## 3 Evolution of the hypothesis

The rationale for using PRP as an adjunctive therapy in eczema lies in its multidimensional regulatory mechanisms, which target core pathological features: inflammation, epidermal barrier dysfunction, and chronic relapse. To evaluate this hypothesis, we examined the therapeutic overlap of PRP across inflammatory skin diseases such as AA and psoriasis, drawing on cross-disease mechanistic parallels to support its potential relevance for eczema.

### 3.1 Core features of eczema and the mechanistic rationale for PRP-based barrier repair

The hallmark of eczema is epidermal barrier dysfunction, characterized by reduced filaggrin (FLG) expression, impaired stratum corneum cohesion, and dysregulated ECM metabolism (23). Enriched with bioactive growth factors, PRP directly addresses these pathological changes (24–27): TGF-β and PDGF promote fibroblast proliferation and ECM synthesis, thereby restoring dermo-epidermal junction integrity (2); EGF and IGF-1 stimulate keratinocyte migration and differentiation, which are essential for reassembling the stratum corneum’s “brick-and-mortar” structure. EGF, in particular, activates keratinocyte differentiation markers such as FLG2 and tight junction proteins including ZO-1 and occluding, thereby reinforcing barrier integrity (28); VEGF enhances microvascularization and improves nutrient delivery to compromised skin.

PRP’s regenerative potential has also been demonstrated in other inflammatory or barrier-deficient dermatoses, such as AA, where it promotes hair follicle regeneration via upregulation of follicular stem cell markers and suppression of perifollicular inflammation (29). Although the specific manifestations of barrier defects differ between eczema and AA, both conditions share a core requirement for epithelial repair—a process directly facilitated by PRP’s growth factor-mediated mechanisms. This mechanistic overlap supports the rationale for using PRP to treat barrier dysfunction in eczema.

### 3.2 Immunomodulatory function: targeting shared pathological T-cell dysregulation across diseases and neuroimmune modulation

Eczema pathogenesis primarily involves an imbalance in the polarization of Th2, Th17, and Th22 cells, along with dysregulated molecular cascades of associated cytokines (30–33). Specifically, IL-4 and IL-13 secreted by Th2 cells bind directly to receptors on keratinocytes and downregulate FLG and tight junction proteins (ZO-1, occludin) via activation of the JAK/STAT6 signaling pathway, thereby compromising the epidermal “brick-and-mortar” structure and increasing TEWL. Th17-derived IL-17 activates the NF-κB pathway, inducing keratinocytes to release pro-inflammatory factors such as IL-6 and TNF-α, which intensify inflammatory infiltration. IL-22 from Th22 cells disrupts keratinocyte proliferation and differentiation balance through STAT3 signaling, resulting in skin thickening and desquamation. As a key pruritogen produced by Th2 cells, IL-31 binds the IL-31RA/OSMR heterodimeric receptor on sensory neurons and activates the JAK/JNK pathway, promoting TRPV1 ion channel phosphorylation (33). This process initiates itch signal transmission and sustains a vicious cycle of itching, scratching, and further barrier disruption.

The immunomodulatory effects of platelet-rich plasma PRP in AA and psoriasis provide molecular evidence supporting its potential intervention in the Th cell axis of eczema. In AA, a Th1/Th17-dominant condition, PRP lowers mRNA expression of Th1/Th17-related pro-inflammatory factors such as IFN-γ and IL-17 in lesions, while increasing the anti-inflammatory factor IL-10 and levels of the regulatory T-cell (Treg) marker FOXP3 (13). This core mechanism relies on PRP-derived TGF-β1, which inhibits dendritic cell maturation and downregulates surface co-stimulatory molecules CD80/CD86, thereby reducing dendritic cell (DC) -mediated activation of pathogenic T cells (13). Similarly, in the Th17-dominant setting of psoriasis, PRP regulates DC-T cell interactions by reducing peripheral blood Th17 cells, suppressing their secretion of IL-17, IL-22, and TNF-α, and enhancing the suppressive capacity of FOXP3+ Treg cells over effector T cells (14, 34). Although eczema is characterized by Th2/Th22 polarization, which differs from the Th profiles in alopecia areata and psoriasis, all three disorders share the pathological features of excessive pathogenic T cell activation and impaired Treg function. This commonality suggests that PRP may restore balance to the Th2/Th17/Th22 axis in eczema by modulating DC-T cell interactions and reinforcing Treg activity.

Beyond T cell immunomodulation, PRP may also disrupt the “itch-inflammation” cycle in eczema via neuroimmune pathways. In models of chronic neuropathic pain, PRP shifts the local microenvironment from a pro-inflammatory to an anti-inflammatory state, producing analgesic effects (20). It has been shown to alleviate pain in tendinosis, tendon injury, rotator cuff tears, osteoarthritis, plantar fasciitis, muscle injuries, and burn-induced neuropathic pain (20). Since the “itch-scratch” cycle in eczema depends on sensory neuron activation mediated by neuropeptides (35), we hypothesize that PRP exerts analogous neuroimmune regulation: it may inhibit IL-31-induced TRPV1 ion channel activation to reduce itch signaling, while concurrently repairing the epidermal barrier and modulating Th2/Th22 cytokine secretion to impede the chronic progression of eczema through multiple complementary pathways.

### 3.3 Antimicrobial activity: addressing S. aureus colonization in eczema through cross-disease infection susceptibility

Eczematous skin is commonly colonized by S. aureus, which exacerbates inflammation through the secretion of superantigens and the formation of biofilms (36). Although S. aureus infections are not typically predominant in psoriasis or AA, the broad-spectrum antimicrobial activity of PRP—both direct and host-mediated—corresponds to the distinct infection risks associated with eczema. PRP acts synergistically with β-lactam antibiotics such as ampicillin or oxacillin to reduce methicillin-resistant S. aureus (MRSA) colony-forming units (CFU) by three logs (21). Mechanistically, PRP activates innate immunity by recruiting neutrophils and increasing reactive oxygen species (ROS) production, thereby enhancing bacterial clearance (37, 38). These mechanisms may help alleviate the adverse effects of secondary infections on eczema progression.

### 3.4 Regulation of inflammation and tissue regeneration: the dual role in bridging chronic eczema inflammation and repair

Eczema is characterized by the “itch-scratch-inflammation” cycle, where chronic inflammation damages sensory nerves, further exacerbating itching and tissue damage. The dual role of PRP in inflammation control and tissue healing has been observed in conditions such as AA (13, 26), psoriasis (14), and rheumatoid arthritis (39), suggesting its potential to specifically interrupt this cycle. In AA, PRP accelerates hair follicle regeneration by promoting angiogenesis and reducing perifollicular fibrosis (13). In psoriasis, PRP alleviates keratinocyte hyperproliferation and plaque inflammation by inhibiting IL-17 and TNF-α (14).

For eczema, PRP may suppress IL-31 (itch-related) and IL-1β (neuro-sensitization -related) (13, 30–32) through the action of its released or activated endogenous anti-inflammatory factors, such as TGF-β1 and IL-10 (40). Meanwhile, its pro-regenerative factors (e.g., VEGF, EGF) can repair nerve endings and epidermal structure—thereby breaking the “itch-scratch” cycle.

### 3.5 Enhanced skin hydration: a synergistic adjunct to barrier restoration

Eczematous skin is characterized by xerosis resulting from depleted natural moisturizing factors (NMFs) and inadequate sebum production, which further aggravates barrier dysfunction (23, 41). Although PRP itself lacks humectant properties, it indirectly enhances skin hydration by stimulating keratinocyte proliferation and migration. Through the restoration of barrier integrity—including the recovery of FLG expression and ECM synthesis—PRP mitigates TEWL, a key indicator of cutaneous dryness in eczema (42, 43). The barrier-repairing efficacy of PRP has also been observed in other eczematous and xerotic dermatoses. For instance, PRP combined with biomaterials such as electrospun fibers has been shown to improve skin hydration by enhancing barrier function in psoriasis, which presents with scaling and dryness, and in AA, which involves scalp dryness (44, 45).

Overall, the use of PRP in eczema aligns with its established mechanistic basis across inflammatory skin disorders. Eczema shares core pathological features with conditions like psoriasis and AA, including immune dysregulation (T-cell skewing, Treg dysfunction), epithelial barrier defects (aberrant keratinocyte differentiation, ECM imbalance), chronic inflammation (cytokine-mediated tissue damage), and increased microbial susceptibility (S. aureus colonization in eczema; opportunistic infections in psoriasis/AA). While eczema is primarily driven by Th2/Th17/Th22 pathways—contrasting with the Th1/Th17 polarization seen in psoriasis or AA—PRP’s multifunctional profile (immunomodulation, growth factor secretion, antimicrobial effects) enables it to concurrently target these shared pathological elements. This positions PRP as a trans-disease regulator capable of addressing the interplay of immune, barrier, and microbial disturbances in eczema.

However, eczema encompasses heterogeneous subtypes with distinct pathophysiology, necessitating subtype-specific mechanistic consideration. AD is rooted in genetic epidermal barrier defects (e.g., FLG mutations), causing stratum corneum disorganization and increased TEWL (46). Allergic contact dermatitis involves allergen-mediated type IV hypersensitivity reactions, while irritant contact dermatitis results from direct keratinocyte damage by chemical/physical irritants without prior sensitization (47, 48). Nummular/asteatotic eczema is linked to lipid metabolic abnormalities and environmental dryness (49).

Consequently, PRP demonstrates subtype-specific therapeutic effects in eczema (**Table 1**): in AD, it may repair genetic defects by upregulating FLG via EGF/TGF-β, enhancing keratinocyte differentiation to restore barrier “brick-and-mortar” structure while alleviating chronic dryness via VEGF-improved microcirculation (42, 43); for allergic contact dermatitis, its anti-inflammatory actions (suppressing IL-1β/TNF-α) mitigate acute flares, and PDGF accelerates epidermal regeneration; in irritant contact dermatitis, PRP promotes repair via PDGF and dampens acute barrier disruption with anti-inflammatory factors like IL-1Ra; and for nummular/asteatotic eczema, it targets lipid metabolism to restore barrier function, with its TEWL-reducing moisturizing effect particularly beneficial in dry environments.

**Table 1:** Eczema subtypes: barrier disruption mechanisms and PRP’s potential targeted effects.

Eczema subtype	Core barrier defect	Key pathological mechanisms	PRP’s potential key actions	Clinical impact
Atopic Dermatitis (AD)	Genetic defects (e.g., FLG mutations) → disrupted stratum corneum “brick-and-mortar” structure (46)	Elevated pH, microbial dysbiosis, Th2/Th22-driven inflammation (IL-4, IL-13, IL-31) → ↑TEWL	Upregulates FLG expression; enhances keratinocyte differentiation; improves microcirculation → repairs barrier, reduces dryness (42, 43)	Restores structural integrity; mitigates chronic dryness and susceptibility to triggers
Allergic Contact Dermatitis	Localized barrier breach at antigen penetration sites (47)	Type IV hypersensitivity → Th1/Th17 activation; acute inflammation (IL-1β, TNF-α)	Suppresses IL-1β/TNF-α; accelerates epidermal regeneration → shortens recovery post-insult (13, 30–32)	Reduces acute flares; accelerates healing of antigen-induced damage
Irritant Contact Dermatitis	Acute physical/chemical damage to stratum corneum (48)	Direct keratinocyte cytotoxicity → barrier collapse; localized inflammation	Promotes rapid epidermal repair via PDGF; dampens inflammation → mitigates acute barrier failure (2)	Shortens repair cycle; prevents acute barrier breakdown
Nummular/Asteatotic Eczema	Lipid metabolism defects → xerosis (49)	Environmental dryness → lipid deficiency; impaired NMF production → ↑TEWL	Stimulates keratinocyte proliferation/lipid synthesis; reduces TEWL → repairs lipid-associated barrier defects (28)	Critical for managing dryness-triggered flares in low-humidity environments

→ means “then” or “next”.

↑ means “to intensify” or “to increase”.

## 4 Testing the Hypothesis

To validate the proposed “multi-targeted, low-side-effect” intervention strategy for PRP in eczema, the experimental framework can be organized around a “mechanism elucidation–translational efficacy” axis, structured as a “validation target experiment type” correspondence system (**Table 2**) (50–63).

**Table 2:** Validation dimensions and experimental framework for PRP in eczema.

Validation dimension	Experimental models	Interventions	Assessment metrics
Growth Factor-Mediated Skin Repair	*In vitro*: Human lesional/non-lesional epidermal KCs cultured under IL-4/IL-13-induced eczema microenvironment *In vivo*: Murine eczema model (51)	*In vitro*: PRP-conditioned medium, recombinant TGF-β alone, blank control *In vivo*: Topical PRP injection (lesional area) or cream; controls: saline, topical steroids	*In vitro*: KCs proliferation (Ki67), differentiation (FLG, loricrin, keratins 1/10 (50)), ECM synthesis (collagen I/III), antioxidant capacity (SOD/MDA), melanin content *In vivo*: TEWL, skin hydration, histology (HE/CD31 staining), pruritus behavior (scratch counts)
Immunomodulatory Function	*In vitro*: Peripheral blood mononuclear cells (PBMCs) from eczema patients (differentiated into Th1/Th2/Th17/Treg) (52) *In vivo*: Murine eczema model	*In vitro*: PRP co-culture with PBMCs (gradient doses); controls: PBS, IL-10-neutralizing antibody *In vivo*: Intradermal PRP + Treg infusion; controls: PRP-only or Treg-only	*In vitro*: Cytokines (IFN-γ, IL-4, IL-13, IL-17, IL-10), Treg proportion, signaling pathways (NF-κB/STAT3/STAT5 phosphorylation) *In vivo*: Serum IgE/IL-4/IL-13, skin lymphocyte/Treg infiltration, inflammatory cell counts (CD3+/mast cells)
Antimicrobial Activity	*In vitro*: S. aureus (ATCC strains) and clinical MRSA isolates (from eczema patients) *In vivo*: Murine skin inoculated with S. aureus suspension	*In vitro*: PRP (various concentrations), PRP + β-lactam antibiotics, antibiotic monotherapy, PBS *In vivo*: Topical PRP; controls: saline, mupirocin ointment	*In vitro*: MIC/MBC, biofilm inhibition (crystal violet staining), antimicrobial peptide release *In vivo*: Bacterial load, cytokines (IL-1β/TNF-α), neutrophil infiltration
Inflammation Regeneration Crosstalk	*In vitro*: Dorsal root ganglion (DRG) neurons + dermal fibroblasts co-culture (simulating neuro-immune interactions) (58) *In vivo*: Murine chronic eczema model	*In vitro*: PRP supernatant treatment; controls: PBS, NGF *In vivo*: Weekly PRP injection (4 weeks); controls: saline, calcineurin inhibitors	*In vitro*: Neuronal activity, IL-31/TGF-β1, proteomics *In vivo*: Cytokines (IL-1β/TNF-α/IL-31), collagen deposition (Masson staining), pruritus-related scratching behavior
Skin Hydration Enhancement	*In vitro*: Tape-stripped stratum corneum from eczema patients/healthy controls *In vivo*: Murine chronic eczema model	*In vitro*: PRP-treated corneocytes vs. saline/positive control (ceramide cream) *In vivo*: Weekly PRP injection (4 weeks); controls: saline	*In vitro*: Corneocyte hydration, FLG/Claudin-1 expression *In vivo*: Dynamic TEWL, scanning electron microscopy (SEM) of stratum corneum
Clinical Validation (Definitive Proof)	Patients meeting Hanifin-Rajka criteria (62), EASI ≥16, refractory to conventional therapy (63)	*Intervention*: Weekly PRP injection (8 weeks) + moisturizer *Control*: Saline injection + moisturizer	Primary endpoints: EASI, visual analog scale (VAS), Insomnia Severity Index (ISI), Dermatology Life Quality Index (DLQI), and Patient-Oriented Eczema Measure (POEM) scores (62) Secondary endpoints: Serum IL-4/IL-13/TGF-β1, TEWL/FLG, safety (local/systemic adverse events)

At the basic research level, *in vitro* cell models such as IL-4/IL-13-stimulated keratinocytes (KCs) (50) will assess PRP’s direct barrier repair capacity via growth factors, while *in vivo* animal models like chronic eczema mouse models (51) will visually demonstrate its overall improvement of eczema phenotypes, including pruritus, erythema, and desquamation. For immune dysregulation, *in vitro* co-culture of immune cells will evaluate PRP’s suppression of Th2/Th17 pro-inflammatory cytokines (IL-4, IL-17) and enhancement of Treg anti-inflammatory functions (FOXP3, IL-10) (13); *in vivo* experiments will further examine its modulation of systemic serum IgE and local cutaneous inflammatory infiltration mediated by CD3^+^ T cells and mast cells (53). Antimicrobial effects will be tested through *in vitro* antibacterial assays (54), complemented by *in vivo* measurements of bacterial load and inflammatory mediators such as IL-1β and TNF-α, to confirm PRP’s ability to inhibit S. aureus colonization and prevent infection-driven exacerbation (55–57). For inflammation regulation and tissue regeneration, neuro-immune co-culture models and chronic wound repair models (58, 59) will investigate PRP’s capacity to disrupt the “itch–scratch–inflammation cycle” by suppressing chronic inflammation and promoting collagen deposition. Skin hydration improvement will be evaluated using *in vitro* stratum corneum hydration assays and *in vivo* hydration monitoring via dynamic TEWL and scale morphology observation (60, 61). Finally, clinical randomized controlled trials (RCTs) will translate these mechanistic insights into efficacy outcomes using core endpoints such as the Eczema Area and Severity Index (EASI) score and recurrence rate (62, 63), while also evaluating safety parameters including local adverse reactions and systemic infection risk to ensure PRP’s scientific validity and clinical feasibility. The entire validation framework is closely aligned with eczema’s complex pathophysiological network involving barrier dysfunction, immune dysregulation, infection, and inflammation, thereby providing comprehensive experimental support for the “repair-oriented” therapeutic strategy.

For the hypothesis to be supported, results across dimensions must satisfy the following criteria:

Mechanistic level: PRP significantly upregulates growth factor expression in eczema models, promotes KCs proliferation and ECM synthesis (barrier repair); suppresses Th2/Th17 cytokines (IL-4, IL-17) and upregulates Treg proportion and IL-10 (immunomodulation); reduces S. aureus load and biofilm formation (antimicrobial activity); decreases IL-1β, TNF-α, and NGF (inflammation and neuroregulation); and improves stratum corneum hydration and TEWL (hydration enhancement).

Animal level: PRP-treated eczema mice show significantly reduced skin inflammation scores, scratching frequency, and TEWL compared to controls, with histological analysis revealing normalized epidermal thickness and organized collagen structure.

Clinical level: The PRP trial group exhibits significantly higher EASI response rates, greater improvement in pruritus Visual Analog Scale (VAS) scores, and lower recurrence rates than the placebo group; mechanistic markers such as elevated serum IL-10 and reduced TEWL correlate positively with clinical efficacy.

## 5 Discussion

Current eczema management has evolved into a multimodal intervention framework encompassing topical corticosteroids, calcineurin inhibitors, immunosuppressants, biologics, and JAK inhibitors (4–6, 64). However, these existing approaches fail to fully address the unmet needs of eczema management. Conventional eczema therapies exhibit inherent limitations: although topical corticosteroids and calcineurin inhibitors such as tacrolimus swiftly suppress inflammation, they do not restore barrier integrity and can further impair barrier function by inhibiting epidermal proliferation (65). Similarly, biologics like dupilumab, despite precise inhibition of the Th2 pathway (66), target only a single immune mechanism and neglect other pathological elements such as Th17 hyperactivation and microbial infections, while also posing risks of drug resistance. PRP’s multi-targeted approach corresponds directly to the multifactorial pathogenesis of eczema, presenting a promising strategy to overcome these therapeutic constraints. Although platelets are primarily recognized for their role in hemostasis, emerging evidence indicates their involvement in systemic inflammatory processes, including cutaneous inflammation (67). Preliminary investigations have demonstrated that PRP can ameliorate symptoms in certain inflammatory skin disorders, such as psoriasis and AD (68). This hypothesis posits that PRP may exert multidimensional core value in eczema treatment: Its growth factor-rich composition directly repairs the epidermal barrier—for instance, through enhanced keratinocyte proliferation and ECM synthesis—thus overcoming the principal limitation of conventional anti-inflammatory treatments that do not restore barrier integrity. By modulating key T-cell subsets such as Th2 and Th17 and regulating associated cytokines, PRP promotes broad-spectrum immune equilibrium, surpassing the narrow targeting of biological agents. Its inherent antimicrobial properties, including the potentiation of neutrophil function, disrupt the S. aureus-driven cycle of infection and inflammation, thereby reducing reliance on antibiotics. The dual capacity to suppress pro-inflammatory cytokines like IL-1β while stimulating fibroblast proliferation disrupts the itch–scratch cycle and alleviates chronic tissue damage. Furthermore, PRP enhances skin hydration over the long term by restoring barrier function and encouraging ECM synthesis, thus minimizing environmental triggers. In summary, this framework proposes that PRP acts systematically on eczema pathophysiology via five core mechanisms: growth factor-mediated repair, immunomodulation, antimicrobial activity, synergy between anti-inflammatory and regenerative processes, and hydration improvement. This multitargeted strategy aligns more effectively with the complex and chronic nature of eczema than do conventional single-target therapies. Multidimensional experimental validation may systematically confirm that PRP, through integrating these mechanisms, offers effective eczema management, thereby supporting both the scientific and clinical value of this hypothesis. If future large-scale randomized controlled trials confirm its efficacy in chronic recurrent or steroid-resistant eczema, PRP could transition from an adjunctive therapy to a critical component of comprehensive eczema management. This would drive a paradigm shift in clinical practice—from “symptom control” to “pathological repair + functional reconstruction”—ultimately offering patients more personalized and sustainable treatment options.

Despite the compelling theoretical foundation for PRP’s multi-targeted mechanisms, several critical challenges must be addressed before clinical translation can proceed.

First, optimal administration routes—such as topical application versus intralesional injection, or monotherapy versus combination therapy—require clarification. This necessitates consideration of the heterogeneous needs of eczema patients across disease severities, alongside exploration of PRP’s potential as a standalone treatment or in combination with existing therapies such as dupilumab, JAK inhibitors, or standard topical regimens. Topical application of PRP is limited by the poor skin penetration of its large molecular components, though low-energy laser-assisted delivery has been shown to enhance absorption (69). Novel delivery systems, including recombinant collagen-based transdermal platforms, have been developed to improve contact and reduce drug waste (70). Although intralesional injection delivers PRP directly to the target site, it carries risks of pain and infection. Since eczema lesions are often colonized by Staphylococcus aureus and susceptible to spontaneous infection, strict preventive measures—such as thorough skin disinfection, stringent aseptic technique, and antimicrobial pretreatment when indicated—are essential during PRP administration to minimize infectious complications. Second, standardization of PRP preparation is paramount. While both single-spin and double-spin centrifugation techniques yield PRP, significant variations may exist in platelet concentration, bioactive molecule composition, and release kinetics (71). These discrepancies directly impact therapeutic reproducibility, underscoring the urgent need for unified protocols. Third, a deeper mechanistic understanding is needed to define the dose-response relationships of AD-associated growth factors and to tailor strategies to individual skin microenvironments. For example, clarifying how specific growth factor thresholds correlate with symptom improvement in mild versus severe AD could inform precision dosing that moves beyond broad “low versus high” guidelines toward target-specific bioactivity levels. Personalized approaches must also account for key microenvironmental variables: *S. aureus* burden, distinct Th2/Th17/Th22 cytokine signatures (IL-4, IL-13, IL-31 dominance), and barrier defects such as filaggrin or ceramide depletion all modulate PRP responsiveness. This knowledge will strengthen the scientific rationale for PRP use, ensuring that interventions are grounded in mechanistic insight rather than empirical observation and are better aligned with AD’s inherent biological heterogeneity.

## 6 Conclusion

This hypothesis proposes that PRP systematically modulates the core pathological processes of eczema—barrier disruption, immune dysregulation, chronic inflammation, infection, and dryness—via synergistic mechanisms. These include growth factor-induced barrier restoration, immunomodulation, antimicrobial action against S. aureus, disruption of the damage–inflammation cycle through inflammation–regeneration coupling, and improved skin hydration. Its core significance lies in transcending the limitations of conventional suppressive therapies, which merely alleviate symptoms, and offering a more efficient, safe adjunct strategy for eczema management. Future research should prioritize systematically validating the hypothesis’s scientific and clinical value through multidimensional experimental approaches.
